# Exploring how surface treatments affect the bond stability of lithium disilicate ceramic to resin cement during try-in

**DOI:** 10.1590/0103-644020256383

**Published:** 2025-08-11

**Authors:** Rafaela Oliveira Pilecco, Renan Vaz Machry, Amanda Maria de Oliveira Dal Piva, João Paulo Mendes Tribst, Cornelis Johannes Kleverlaan, Rafael R. Moraes, Gabriel Kalil Rocha Pereira

**Affiliations:** 1 Department of Conservative Dentistry, Universidade Federal do Rio Grande do Sul (UFRGS), Porto Alegre, Rio Grande do Sul, Brazil; 2 Department of Restorative Dentistry, Faculty of Dentistry, Universidade Federal de Minas Gerais(UFMG), Belo Horizonte, Minas Gerais, Brazil; 3 Department of Dental Materials Science, Academic Centre for Dentistry Amsterdam (ACTA), Universiteit van Amsterdam and Vrije Universiteit, Amsterdam, North Holland, The Netherlands; 4 Department of Reconstructive Oral Care, Academic Centre for Dentistry Amsterdam (ACTA), Universiteit van Amsterdam en Vrije Universiteit, Amsterdam, The Netherlands; 5 Faculty of Dentistry, Universidade Federal de Pelotas(UFPel), Pelotas, RS, Brazil; 6 Post-Graduate Program in Oral Sciences (Prosthodontics Unit), Faculty of Dentistry, Universidade Federal de Santa Maria(UFSM), Santa Maria, Rio Grande do Sul, Brazil.

**Keywords:** Ceramic, Cleaning, Dental bonding, Hydrofluoric acid, Resin cement, Silane, Surface treatment, Try-in

## Abstract

This study evaluated how different surface treatments affect the bond strength of resin cement to lithium disilicate ceramic subjected to try-in. Lithium disilicate (IPS e.max CAD) slices were randomly assigned to 5 experimental groups based on surface treatment protocols with 5% hydrofluoric acid (HF) and silane (SIL) application timing: HF PRE + SIL POST (HF before and SIL after), HF SIL PRE (both HF and SIL before), HF SIL POST (both HF and SIL after), HF SIL PRE + SIL POST (SIL reapplied after), and HF SIL PRE + HF SIL POST (re-treated). Two control groups were included: CTRL+ (no try-in) and CTRL- (no cleaning step after try-in). Resin cement cylinders (n= 56) were bonded and tested for microshear bond strength after 24 hours (baseline) or after 210 days + 25,000 thermal cycles. At baseline, no significant differences were found. After aging, the re-treated groups had the highest bond strength, while the control groups had the lowest. Intermediate results were observed in HF SIL PRE, HF SIL POST, and HF PRE + SIL POST. Try-in remnants were found on HF SIL PRE, and re-etching led to significant matrix dissolution. Post-try-in silane application created a surface layer on the etched ceramic. Cleaning after try-in is crucial for long-term adhesive behavior, regardless of the surface treatment protocol. Applying silane before try-in acts as a protective layer while reapplying silane after try-in further enhances adhesive stability.

## Resumo

Este estudo avaliou como diferentes tratamentos de superfície afetam a resistência de união do cimento resinoso à cerâmica de dissilicato de lítio submetida à pasta try-in. Fatias de dissilicato de lítio (IPS e.max CAD) foram distribuídas aleatoriamente em 5 grupos experimentais com base nos protocolos de tratamento de superfície com ácido fluorídrico (HF) a 5% e na aplicação do silano (SIL) em diferentes momentos: HF PRE + SIL POST (HF antes e SIL depois), HF SIL PRE (HF e SIL antes), HF SIL POST (HF e SIL depois), HF SIL PRE + SIL POST (SIL reaplicado depois) e HF SIL PRE + HF SIL POST (retratado). Dois grupos controle foram incluídos: CTRL+ (sem pasta try-in) e CTRL- (sem etapa de limpeza após a prova). Cilindros de cimento resinoso (n= 56) foram fixados e testados quanto à resistência de união por microcisalhamento após 24 horas (baseline) ou após 210 dias + 25.000 ciclos térmicos. No baseline, não foram encontradas diferenças significativas. Após o envelhecimento, os grupos retratados apresentaram a maior resistência de união, enquanto os grupos controle tiveram os menores valores. Resultados intermediários foram observados nos grupos HF SIL PRE, HF SIL POST e HF PRE + SIL POST. Resíduos da pasta try-in foram encontrados no grupo HF SIL PRE, e o recondicionamento levou a uma dissolução significativa da matriz. A aplicação de silano após a pasta try-in criou uma camada superficial na cerâmica condicionada. A limpeza após a pasta try-in é fundamental para o comportamento adesivo a longo prazo, independentemente do protocolo de tratamento de superfície. A aplicação de silano antes da pasta try-in atua como uma camada protetora, enquanto sua reaplicação após a pasta try-in melhora ainda mais a estabilidade adesiva.

**Figure:**
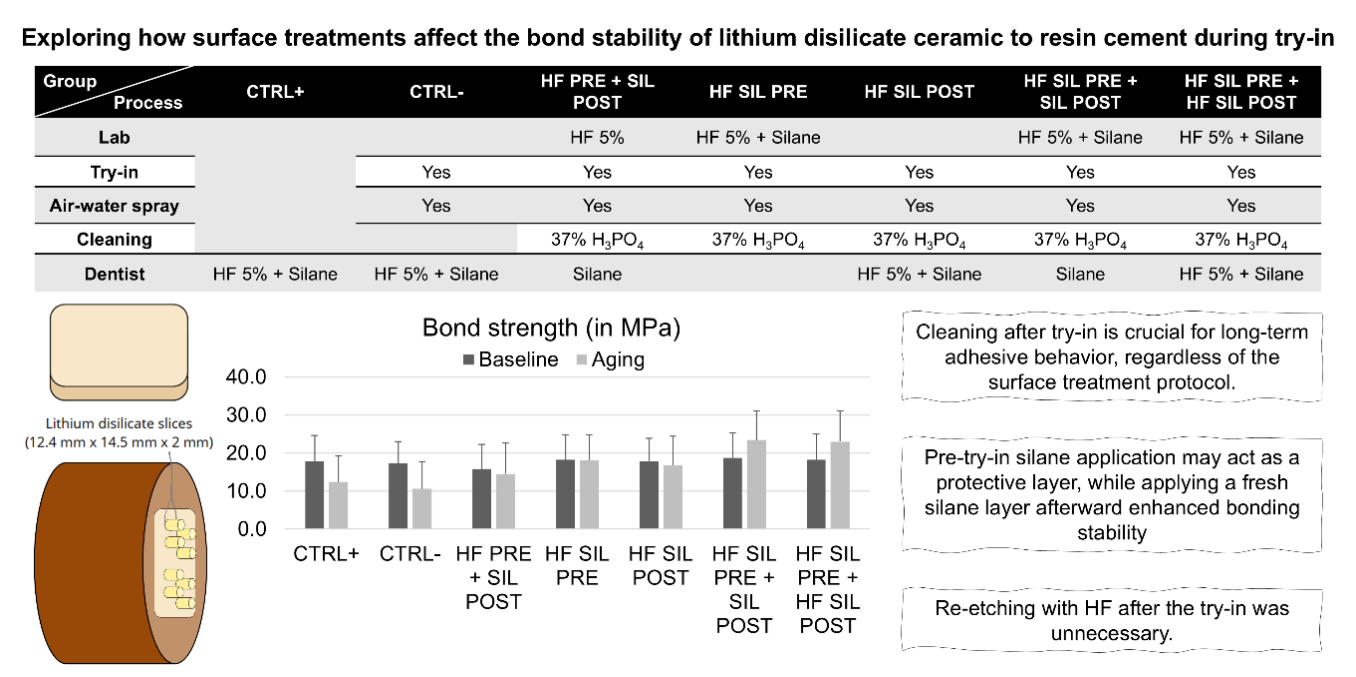

## Introduction

Dental monolithic glass ceramic restorations have surpassed classical and conventional fixed partial dentures, which consist of porcelain with an underlying metallic infrastructure, due to their biocompatibility, esthetics, mechanical properties, and bonding ability to the tooth structure of most monolithic materials such as glass-ceramics ^1^. The adhesion of these materials is achieved in a strong, reliable, and durable manner using resin cement to bond an etchable ceramic ^2^. This characteristic is present due to a vitreous matrix susceptible to etching during the surface treatment ^2^. Among these, lithium disilicate ceramics excel, thanks to needle-like reinforcing crystals dispersed in the vitreous matrix, enhancing their mechanical performance over most glass ceramics ^3^. Lithium disilicate also demonstrates optimal clinical longevity (with a survival rate of 83.5% after 10 years) and superior esthetic appearance by mimicking the optical characteristics of natural dentition ^4^.

One of the clinical indications of this material is thin restorations, such as veneers, which enable higher light transmission through the restoration, achieving a more natural appearance ^1^. It is known that the clinical try-in is an important procedure with thin restorations as it permits the visualization of the final esthetic appearance ^5^ ^,^ ^6^. To improve the esthetic outcome, especially considering the possibility of various substrate shades, the dentist may use try-in pastes to simulate the interaction between the restorative material and the resin cement with the underlying tooth structure ^5^ ^,^ ^6^. However, previous studies have shown that remnants of the try-in paste may be found on lithium disilicate surfaces even after cleaning ^7^ ^,^ ^8^, which can impair the bond strength between the restorative material and resin cement ^8^. A simple way to remove these contaminants from the lithium disilicate surface is the active application of phosphoric acid ^7^ ^,^ ^8^.

Due to the minimized mechanical retention of thin restorations, bonding is a high-risk and essential step to ensure a strong link between the ceramic, resin cement, and tooth structure, aiming to form a consolidated relationship ^5^. For this purpose, the gold standard surface treatment for lithium disilicate is hydrofluoric acid (HF) etching, followed by a silane coupling agent ^2^ ^,^ ^9^ ^,^ ^10^. The HF etching partially removes the vitreous matrix, enabling micromechanical and intimate contact with the resin cement tags ^10^ ^,^ ^11^ ^,^ ^12^ ^,^ ^13^. The silane coupling agent also acts on silica-based restorations by chemically interacting with resin materials, forming a functional monolayer of siloxane bonds ^14^ ^,^ ^15^ ^,^ ^16^. This makes the ceramic surface more prone to bond ^17^ ^,^ ^18^ ^,^ ^19^. Therefore, this surface treatment enables optimal bonding and mechanical performance for lithium disilicate ceramic when properly done.

In this sense, one of the basic principles of a proper bonding procedure is achieving a clean surface ^20^ ^,^ ^21^. Ideally, the dentist should perform all surface treatments after the clinical try-in ^9^. However, it is important to consider that etching the piece with HF may be done by the laboratory before sending it to the dentist due to HF’s toxicity and corrosive potential, as its use is restricted in the dental office in some countries ^22^. In this case, performing the try-in before surface etching may increase the adhesion of contaminating agents on the prosthetic piece due to the resulting increase in surface roughness ^23^, and even so, HF can be reapplied after try-in in some countries, re-etching may result in more aggressive glass-matrix removal without improving adhesion ^13^. Furthermore, a previous study observed that applying silane before the clinical try-in may act as a protective layer against surface contamination ^24^. However, it is still unknown whether the silane layer remains stable or if there is a need to reapply any step of the surface treatment protocol when cleaning methods were used to remove the try-in-paste residues.

Previous research has focused either on surface treatments alone or on cleaning methods after try-in, but none have investigated the timing of surface treatment protocols in the context of the try-in procedure. Thus, this in vitro study aimed to investigate the impact of the timing protocol of the surface treatment with HF etching and silane application on the short and long-term adhesion of lithium disilicate ceramic to resin cement when a try-in paste is used. The first null hypothesis was that the surface treatment protocol would not affect the bond strength of the lithium disilicate ceramic to resin cement. The second hypothesis was that the aging protocol would negatively influence the bond strength.

## Materials and methods

### Study design

**Figure 1:**
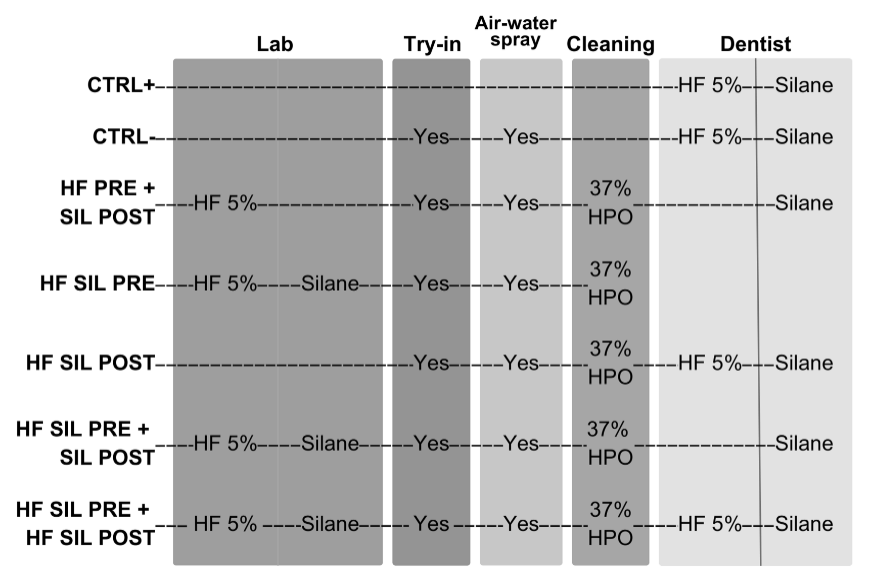
A flowchart of the study design depicts the stages involved in testing different experimental groups: HF 5%, 5% hydrofluoric acid, 37% HPO, and 37% phosphoric acid.

This *in vitro* study involved 7 groups, categorized based on 2 factors: (i) surface treatment protocol and (ii) aging (Figure 1). The main response variable was micro shear bond strength (µSBS) between resin cement and ceramic (Figure 1). A sample size calculation was carried out using data from a pilot study (n= 12) in G*power v. 3.1.9.7, with the following parameters: effect size f= 0.54, α= 00.05, and power= 0.80. This resulted in a minimum of 54 resin cement cylinders in the µSBS test. To account for the potential loss of resin cement cylinders during the bonding process, two additional cylinders were added to each group, making a total of 56 specimens per group.

### Specimen fabrication

Prefabricated lithium disilicate CAD-CAM blocks (IPS e.max CAD, Ivoclar) were cut into rectangular blocks (12.4 mm x 14.5 mm x 2 mm) using a cutting machine (Isomet 1000, Buehler) with constant water-cooling. The surface topography was standardized using a polishing machine (EcoMet/AutoMet 250, Buehler) with silicon carbide (SiC) papers (#400-, #600-, and #1200-grit; Norton). Subsequently, an in-lab simulation protocol was applied to the bonding surface with #60-grit size SiC paper by a calibrated operator (R.O.P.) using digital pressure, moving the sample onto the SiC paper for 15 seconds on each axis of the specimen (*x* and *y*) to achieve a similar topography to CAD-CAM milled ceramics ^8^. The slices were then crystallized according to the manufacturer's recommendation: initial temperature of 403 °C at a rate of 90 °C/min to 820 °C and then increasing the temperature at a rate of 30 °C/min to a final temperature of 840 °C held for 1.5 minutes (Vacumat 6000 MP, VITA Zahnfabrik). A vacuum was applied between 450 °C and 840 °C. The crystallized samples were fixed in polyvinyl chloride plastic rings and embedded with acrylic resin (JET Clássico), exposing the bonding surface.

### Bonding procedure

The surface treatment protocol is described in Figure 1. The choice and characteristics of the surface treatments were made according to a previous study ^8^. Before treatment, specimens were cleaned in an ultrasonic bath with 70% isopropyl alcohol for 5 minutes. Surface etching was performed using 5% HF (Condac Porcelana 5%, FGM) for 20 seconds, followed by an air-water spray with distilled water for 30 seconds and an ultrasonic bath with distilled water for 5 minutes. A fresh silane coupling agent (Monobond N, Ivoclar) was actively applied (Monobond N, Ivoclar) for 15 seconds and allowed to react for an additional 45 seconds. Try-in paste (Variolink N try-in paste in white shade, Ivoclar) - composed of glycerine, mineral fillers, and colorants, was applied to the bonding surface of the experimental groups’ specimens using a spatula. The specimens were positioned with the treated surface facing a cleaned glass plate under a constant load of 2.5 N for 5 minutes. The excess paste was removed with an air-water spray with distilled water for 30 seconds. An active application of 37% phosphoric acid (Attaque Gel, Biodinâmica) with a microbrush for 60 seconds was performed, followed by an air-water spray with distilled water for 30 seconds.

Since the bonding procedure took more than one day, the groups were randomly assigned using a simple randomization method (Excel; Microsoft Corp), taking each bonding day into account to minimize the risk of performance bias. After the surface treatments, starch matrices (n= 56) with height= 1 mm and internal diameter= 1.13 mm (Isabela; M. Dias Branco S.A. Indústria e Comércio de Alimentos) were fixed with sticky wax (Lysanda) over the treated ceramic surface. Dual-cure resin cement Variolink N (Base + low viscosity, Ivoclar) was mixed in a 1:1 ratio and inserted into the starch matrices with a spatula. A dental explorer was used to avoid bubble formation in the resin cement. Light-activation was performed for 40 seconds (1200 mW/cm² Radii-Cal, SDI). Specimens were kept under distilled water at 37 ºC for 24 hours in separate sealed pots. The starch matrices were then carefully removed, and the resin cement cylinders were individually evaluated to ensure no bubbles, defects, or voids were present. Any irregularities led to the exclusion of the resin cement cylinder, and the specimen was replaced. Half of the resin cement cylinders of each ceramic slice were tested immediately, while the other half underwent 210 days of storage under distilled water at 37 ºC and were additionally subjected to 25,000 thermal cycles (30 seconds baths in water at 5 ºC and 55 ºC, the transfer time of 5 seconds; 521-6D, Ethik Technology; Nova Ética) ^25^. The distilled water was not renewed during storage.

### Microshear bond strength test and failure analysis

Each slice was placed onto a universal testing machine (EMIC DL-2000, Instron). A wire-loop method was used with a thin stainless-steel wire (diameter=0.2 mm) looped around each resin cement cylinder as close as possible to the ceramic interface to ensure accurate orientation to shear forces. A crosshead speed of 1 mm/minute was set, and the test was conducted until failure. The µSBS (in MPa) was calculated by dividing the load (in Newtons) by the bonded interface area. Following the test, all specimens were analyzed under a stereomicroscope (Stereo Discovery V20, Carl Zeiss, Oberkochen, Germany) at ×50 magnification to determine the failure pattern of each specimen classified as adhesive (more than 50% of the adhesive area) or cohesive (more than 50% of the resin cement cylinder area).

### Surface topography analysis

One additional specimen from each group was analyzed using scanning electron microscopy (SEM) (VEGA-3G, Tescan) with a secondary electron detector to investigate the surface topography and pattern after the surface treatment protocols. The specimens were sputter-coated with gold and analyzed at ×200 and ×5000 magnifications.

### Statistical analysis

All statistical analyses were conducted using SPSS v.21 (IBM Corp). After data distribution was verified using the Shapiro-Wilk test (p> 0.05) and homoscedasticity verified using the Levene test (p> 0.05), a Two-Way Analysis of Variance (ANOVA) with Bonferroni’s *posthoc* test was performed for bond strength only, considering the predominantly adhesive failures. Descriptive statistics were used for failure patterns. SEM topographic images were qualitatively analyzed and focused on characterizing surface topography, including defects, irregularities, and try-in-paste residues.

## Results

The adhesive behavior at baseline and after aging for the different tested groups is presented in Table 1. According to the two-way ANOVA, the aging protocol did not significantly affect bond strength (F= 2.17; p= 0.141). However, the different surface treatments alone (F=16.94; p< 0.01) and when associated with the aging protocol (F=10.90; p< 0.01) significantly influenced bond strength. At baseline, no differences were observed among the groups (p> 0.05). After aging, the re-treated groups (HF SIL PRE + SIL POST and HF SIL PRE + HF SIL POST) exhibited higher bond strength compared to the others, with no significant difference between them. Groups that received surface treatment either before (HF SIL PRE) or after try-in (HF SIL POST) showed intermediate bond strength results, with no difference also to the HF PRE + SIL POST group. No difference was found between the HF PRE + SIL POST group and the control groups (CTRL+ and CTRL-), with the lowest bond strength results after aging.

While the control groups (CTRL+ and CTRL-) presented a significant decrease in bond strengths after aging (30.7% and 38.7%, respectively), the groups that received the conventional surface treatment a single time presented higher reliability, as shown by bond strength stability after aging. In contrast, there was an increase in bond strength for the groups HF SIL PRE+SIL POST and HF SIL PRE + HF SIL POST, which received the reapplication of silane and the re-etching associated with a new silane application after try-in paste application, respectively.

**Figure 2:**
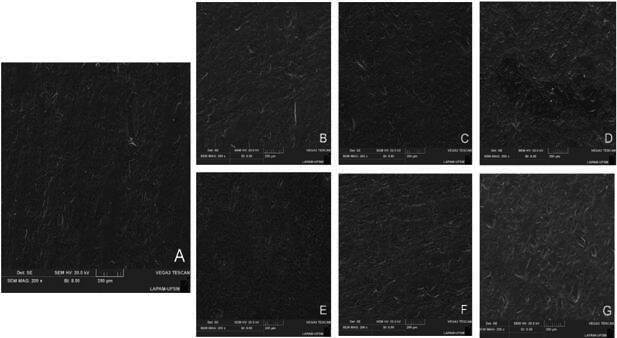
SEM topographic images (×200) of tested groups considering the different surface treatment workflows (A, CTRL+. B, CTRL-. C, HF PRE + SIL POST. D, HF SIL PRE. E, HF SIL POST. F, HF SIL PRE + SIL POST. G, HF SIL PRE + HF SIL POST). There are noticeable try-in remnants in the HF SIL PRE group. Despite this, a comparable macro topography is evident across all tested groups.

**Figure 3:**
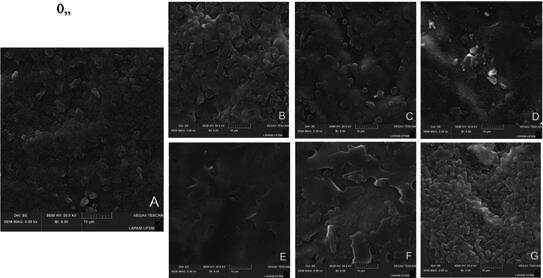
SEM topographic images (×5000) of tested groups surface treatment workflows (A, CTRL+. B, CTRL-. C, HF PRE + SIL POST. D, HF SIL PRE. E, HF SIL POST. F, HF SIL PRE + SIL POST. G, HF SIL PRE + HF SIL POST). The images illustrate distinct topographic features among groups. Re-etched group (G) exhibits pronounced dissolution of the vitreous matrix. Groups, where silane coupling agent was applied after try-in (E and F), display distinct topography by layer on top of the ceramic surface.

Most failures at baseline and after aging were considered predominantly adhesive failures, and all the groups presented similar failure pattern distributions, regardless of surface treatment (Table 2). Pretest failures during the aging procedure were observed only in the HF SIL PRE and HF SIL PRE + HF SIL POST groups (Table 2). The SEM surface topography analysis showed that the macro-topography was similar among the groups (Figure 2). However, try-in remnants were observed in the HF SIL PRE group. Upon closer examination (Figure 3), a pronounced dissolution of the vitreous matrix was visible in the HF SIL PRE + HF SIL POST group. A layer could be seen on top of the etched ceramic in the groups that received a silane application after the try-in (HF SIL POST and HF SIL PRE + SIL POST).

**Table 1:** Mean, standard deviation (SD), and 95% confidence interval (CI) of bond strength (in MPa, n= 56*) at baseline and after aging. Bond strength decreases after aging, as shown in %.

Groups	Baseline		Aging		Average decrease after aging (%)
	Mean (SD)	95% CI	Mean (SD)	95% CI	
CTRL+	17.9 (6.7)^Aa^	16.1 - 19.7	12.4 (6.8)^Cb^	10.5 - 14.2	30.7
CTRL-	17.3 (5.7)^Aa^	15.7 - 18.9	10.6 (7.1)^Cb^	8.7 - 12.6	38.7
HF PRE + SIL POST	15.8 (6.5)^Aa^	14.0 - 17.6	14.5 (8.1)^BCa^	12.3 - 16.7	8.2
HF SIL PRE	18.2 (6.6)^Aa^	16.4 - 20.0	18.1 (6.7)^Ba^	16.3 - 19.9	0.5
HF SIL POST	17.9 (6.0)^Aa^	16.3 - 19.5	16.8 (7.6)^Ba^	14.7 - 18.9	6.1
HF SIL PRE + SIL POST	18.7 (6.6)^Aa^	16.9 - 20.5	23.4 (7.7)^Ab^	21.33 - 25.5	-25.1
HF SIL PRE + HF SIL POST	18.2 (6.8)^Aa^	16.3 - 20.1	23.0 (8.1)^Ab^	20.8 - 25.1	-26.3

HF, Hydrofluoric acid. SIL, Silane.

*Only adhesive failures were considered when calculating bond strength values. Thus, the actual number of specimens in each group is shown in Table 2.

Superscript letters indicate statistically significant differences between the surface treatment protocols in each condition according to Two-Way ANOVA and Bonferroni’s post hoc test (α= 00.05).

Subscript letters indicate statistically significant differences between baseline and aging within the same surface treatment protocol according to Two-Way ANOVA and Bonferroni’s post hoc test (α= 00.05).

**Table 2:** Distribution of the failure modes in bond strength test at baseline and after aging, n= 56

Groups	Baseline		Aging		Pretest failure
	Adhesive	Cohesive	Adhesive	Cohesive	
CTRL+	55 (98.2%)	1 (1.8%)	55 (98.2%)	1 (1.8%)	0 (0.0%)
CTRL-	53 (94.6%)	3 (5.4%)	54 (96.4%)	2 (3.6%)	0 (0.0%)
HF PRE + SIL POST	55 (98.2%)	1 (1.8%)	51 (91.1%)	5 (8.9%)	0 (0.0%)
HF SIL PRE	54 (96.4%)	2 (3.6%)	54 (96.4%)	0 (0.0%)	2 (3.6%)
HF SIL POST	51 (91.1%)	5 (8.9%)	53 (94.6%)	3 (5.4%)	0 (0.0%)
HF SIL PRE + SIL POST	53 (94.6%)	3 (5.4%)	55 (98.2%)	0 (0.0%)	1 (1.8%)
HF SIL PRE + HF SIL POST	53 (94.6%)	3 (5.4%)	53 (94.6%)	3 (5.4%)	0 (0.0%)

HF, Hydrofluoric acid. SIL, Silane.

## Discussion

The surface treatment protocol significantly influenced the bond strength of the lithium disilicate ceramic to a resin cement after aging. Despite no differences being found at baseline, reapplication of silane associated or not with HF re-etching resulted in higher bond strength compared to the other protocols after aging. Thus, the first hypothesis was rejected. Similarly, the second hypothesis was rejected since the aging affected the bond strength.

HF etching followed by a silane coupling agent application is the gold standard surface treatment protocol for lithium disilicate ceramics ^9^. However, some modifications in this protocol might be needed during clinical procedures considering the restoration try-in procedure. As seen herein and supported by previous research ^24^, applying silane before try-in may act as a protective layer against contamination. Therefore, in situations where the lab does HF etching, the dentist should also consider applying a silane layer before clinical try-in. Furthermore, applying a new silane layer after the try-in enhanced the bond strength stability. While concerns have been raised about multiple layers or reapplication of silane after saliva contamination ^14^ as potentially detrimental to adhesion, a fresh silane layer after try-in has been suggested to improve the bond strength stability ^24^, consistent with our findings.

In scenarios where only HF etching is done by the lab before the try-in, there may be a decrease in bond strength stability, considering the slightly worse performance of HF PRE+SIL POST compared to HF SIL PRE. The increased surface roughness caused by HF etching ^23^ may facilitate contaminant retention on the ceramic surface, especially without the protective silane layer before try-in. Although HF SIL PRE showed intermediate bond strength after aging, similar to performing the surface treatment after (HF SIL POST group), remnants of try-in paste were found in SEM images (Figure 2D). However, this did not impair the adhesive behavior.

The beneficial effect of applying a fresh silane layer was found regardless of the re-etching with HF, as both HF SIL PRE + SIL POST and HF SIL PRE + HF SIL POST groups showed similar adhesive behavior. However, considering that re-etching lithium disilicate ceramic could weaken its mechanical strength ^11^, especially for thin restorations due to HF etching’s tridimensional potential to dissolute different regions of the intaglio surface in significant depth ^12^, along with the risk of exposing the dentist and the patient to HF toxicity ^22^, this step may be considered unnecessary if done prior to try-in. Significant topographical modifications were observed in the ceramic after HF re-etching (Figure 2 and 3), resulting in increased surface complexity and roughness. This may have contributed to the higher bond strength in the re-etching group ^13^.

The glass-ceramic nature of lithium disilicate renders it inherently hydrophilic, which is increased by HF etching, as indicated by reduced contact angles compared to non-etched surfaces ^23^ ^,^ ^26^. This hydrophilicity enhances the wettability of silane coupling agents. Nevertheless, proper silanization results in a more hydrophobic layer (i.e., higher contact angle) formed on the ceramic surface ^17^. Our results following surface treatment protocols, particularly the effect of silanization, suggested the successful formation of siloxane bonds in the lithium disilicate surface, even after try-in and cleaning with 37% phosphoric acid. Various factors, including post-silanization protocols, influence the quality and adhesive stability of the bonded interfaces ^2^ ^,^ ^10^ ^,^ ^15^ ^,^ ^16^ ^,^ ^17^. These protocols aim to remove water (a byproduct of the condensation reaction that occurs during siloxane bond formation), solvents, and contaminants ^10^ ^,^ ^17^. As seen herein, the application of 37% phosphoric acid did not compromise the bonding stability of an already silanized ceramic surface, as evidenced by only a 0.5% decrease in bond strength after aging in the HF SIL PRE group. This may be due to the direct access to the ceramic etched and silanized granted by the cleaning with phosphoric acid ^10^, which may have removed the unreacted region of the silane layer (outmost region), maintaining an active film on the ceramic with enhanced resistance to hydrolytic degradation ^16^.

Previous studies have shown no detrimental aging protocol effect on bond strength when using the same resin cement used herein (Variolink), even after aggressive regimes ^18^ ^,^ ^19^. However, this result may depend on the surface treatment applied to the lithium disilicate ceramic, as shown in Table 1. Specifically, retreatment with either silane alone or with both HF and silane resulted in increased bond strength of Variolink to lithium disilicate. On the other hand, our results indicate a significant decrease in bond strength after aging when the cleaning step was omitted (CTRL+ and CTRL- groups). The use of 37% phosphoric acid as a cleaning agent after try-in has been documented as an effective method to enhance the bond strength of lithium disilicate to resin cement ^8^. Our study reinforces the need for thorough cleaning with 37% phosphoric acid after try-in. Therefore, even in scenarios where the ceramic will be etched after try-in paste application, it is important to apply phosphoric acid actively before, considering its potential to act as an organic and acidic dissolution agent ^8^. Both control conditions (CTRL+ and CTRL-) exhibited similar and initially promising adhesive behavior but showed the lowest bond strength stability over long-term aging. It is worth noting that the CTRL+ group, which did not receive a try-in paste application, exhibited worse bond stability compared to the experimental groups. This may be due to two possibilities: either the presence of unknown impurities during specimen fabrication or the absence of phosphoric acid as a cleaning step after HF etching, which is known to leave precipitants on the ceramic surface that may impair adhesive stability ^10^ ^,^ ^20^ ^,^ ^21^. Moreover, all experimental procedures in this study were carried out exclusively by a designated pair of operators adhering meticulously to stringent methodological protocols, thereby reducing any potential for variability in protocol execution.

Besides the *in vitro* study’s inherent limitations, such as the lack of clinical variability, it is important to state that we only evaluated specific materials that may affect the bond strength, as different materials can vary in composition, viscosity, and other properties. For instance, the oily nature of some try-in pastes or the varying viscosities of different cements might lead to different results. Therefore, our findings may not be universally applicable to all types of try-in pastes and resin cements. Additionally, monotonic shear forces applied to simulated specimens do not fully represent the clinical behavior of milled anatomic restorations bonded to the tooth. It is also important to emphasize that pretest failures were not included in the statistical analysis of bond strength, as they occurred in only a few cases (2 in the HF SIL PRE group and 1 in the HF SIL PRE + HF SIL POST group). Therefore, they did not significantly affect the number of samples analyzed. Notably, there is no consensus in the literature regarding the appropriate statistical treatment of pretest failures in bond strength tests involving ceramic substrates. Nevertheless, this study is worthy of attention as it assesses the impact of different surface treatment protocols, simulating a clinical try-in, on the bond strength of lithium disilicate ceramic to resin cement over the long term. Given the common use of try-in paste, it is essential to properly treat the restoration's surface to ensure proper adhesive behavior, highlighting the clinical relevance of this study. Future studies could consider the presence of saliva, another contaminant present during clinical try-in, along with exploring different try-in pastes or alternative cleaning methods.

In conclusion, cleaning the ceramic with 37% phosphoric acid after the try-in phase was crucial for achieving long-term adhesive performance, regardless of the surface treatment. Pre-try-in silane application may act as a protective layer, preserving bond strength even after phosphoric acid cleaning. When etching and silane were performed before the try-in, applying a fresh silane layer afterward enhanced bonding stability. Re-etching with HF after the try-in was unnecessary if cleaning with phosphoric acid and/or reapplying silane had been properly conducted.
